# Dynamics retrieval from stochastically weighted incomplete data by low-pass spectral analysis

**DOI:** 10.1063/4.0000156

**Published:** 2022-08-16

**Authors:** Cecilia M. Casadei, Ahmad Hosseinizadeh, Gebhard F. X. Schertler, Abbas Ourmazd, Robin Santra

**Affiliations:** 1 Institute of Molecular Biology and Biophysics, Department of Biology, ETH Zürich, Zürich, Switzerland; 2 Laboratory of Biomolecular Research, Biology and Chemistry Division, Paul Scherrer Institute, Villigen PSI, Switzerland; 3 University of Wisconsin Milwaukee, Milwaukee, Wisconsin 53201, USA; 4 Center for Free-Electron Laser Science CFEL, Deutsches Elektronen-Synchrotron DESY, 22607 Hamburg, Germany; 5 Department of Physics, Universität Hamburg, 22607 Hamburg, Germany

## Abstract

Time-resolved serial femtosecond crystallography (TR-SFX) provides access to protein dynamics on sub-picosecond timescales, and with atomic resolution. Due to the nature of the experiment, these datasets are often highly incomplete and the measured diffracted intensities are affected by partiality. To tackle these issues, one established procedure is that of splitting the data into time bins, and averaging the multiple measurements of equivalent reflections within each bin. This binning and averaging often involve a loss of information. Here, we propose an alternative approach, which we call low-pass spectral analysis (LPSA). In this method, the data are projected onto the subspace defined by a set of trigonometric functions, with frequencies up to a certain cutoff. This approach attenuates undesirable high-frequency features and facilitates retrieving the underlying dynamics. A time-lagged embedding step can be included prior to subspace projection to improve the stability of the results with respect to the parameters involved. Subsequent modal decomposition allows to produce a low-rank description of the system's evolution. Using a synthetic time-evolving model with incomplete and partial observations, we analyze the LPSA results in terms of quality of the retrieved signal, as a function of the parameters involved. We compare the performance of LPSA to that of a range of other sophisticated data analysis techniques. We show that LPSA allows to achieve excellent dynamics reconstruction at modest computational cost. Finally, we demonstrate the superiority of dynamics retrieval by LPSA compared to time binning and merging, which is, to date, the most commonly used method to extract dynamical information from TR-SFX data.

## I. INTRODUCTION

**FIG. 1.**
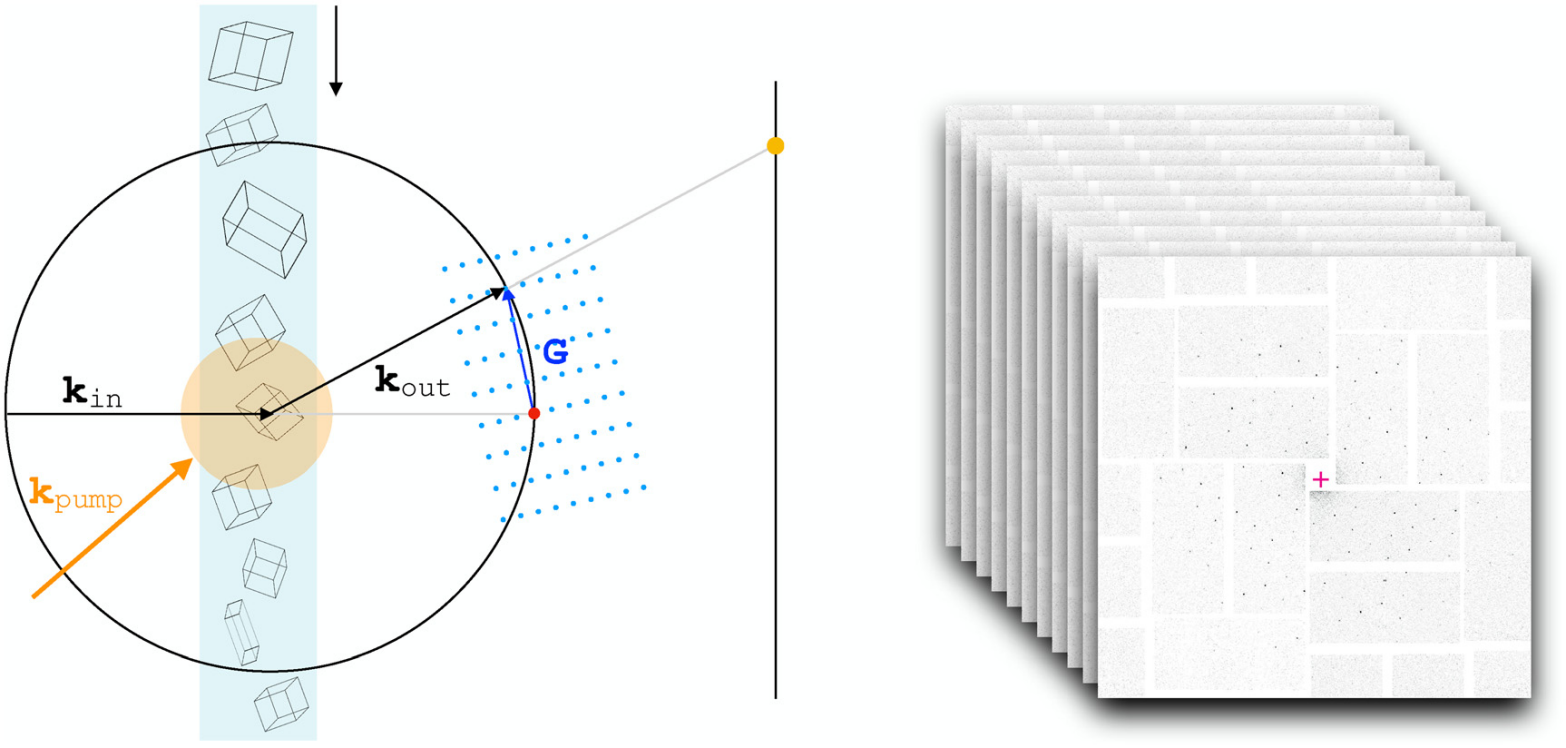
Schematic representation of the time-resolved serial crystallography experiment. The microcrystals are brought into the interaction region by a continuous flow of a viscous medium. Individual crystals are probed by an X-FEL pulse at a certain time delay after optical pumping. The diffraction condition $k_{\text{out}}-k_{\text{in}}=G$, with G a reciprocal lattice vector, is represented graphically by the diffraction sphere construction.^10^ A dataset is composed of an ensemble of frames, each recording a diffraction pattern from an individual crystal in a random orientation.

Time-resolved serial femtosecond crystallography (TR-SFX) has emerged as a prominent technique for investigating the dynamics of light-sensitive macromolecules with atomic spatial resolution at ultrafast timescales.^1–4^ In a typical experiment, a laser pulse pumps the molecules into an excited state. An x-ray pulse from an X-ray Free-Electron Laser (X-FEL) probes the system a certain time delay—typically in the femtosecond to picosecond range—after photo-excitation. Microcrystals of the protein of interest, embedded in a viscous medium, are delivered into the interaction region through a continuous flow. The experiment is carried out in a serial fashion. Since the interaction with the X-FEL beam is destructive, each microcrystal gives rise to up to one diffraction pattern, from a specific, random orientation (Fig. 1).

**FIG. 2.**
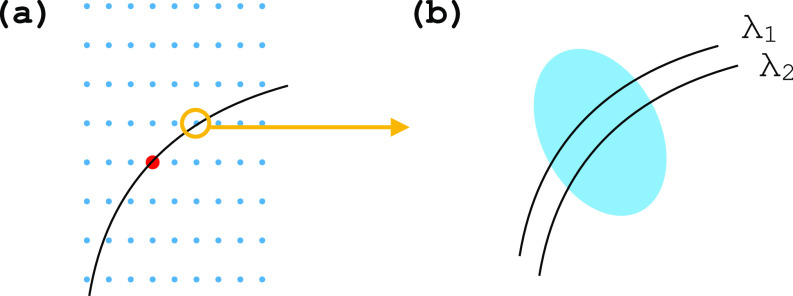
Schematic representation of data incompleteness and partiality. (a) The diffraction condition is satisfied at the intersection between the reciprocal lattice, whose orientation is determined by the crystal's, and the diffraction sphere, with radius $\left|k_{\text{in}}|=|k_{\text{out}}\right|$. Most reciprocal lattice points are unmeasured in an individual frame. This determines the incompleteness of the data. (b) Due to the finite size of the crystal and lattice disorder, the reciprocal lattice regions that can give rise to constructive interference are not vanishingly small, but can rather be modeled as three-dimensional ellipsoids. The spectral distribution of the incident X-FEL beam can be accounted for by attributing a finite thickness to the scattering sphere (scattering shell). In this model, diffraction arises from the volumes at the intersection between reciprocal space ellipsoids and the scattering shell,^11^ so that the resulting partial intensities are, in general, smaller and not representative of the full intensities that would arise from an infinite and perfect crystal.

Under typical experimental conditions, only a small fraction of the reciprocal lattice points within the accessible resolution range fulfill the elastic scattering condition for a specific crystal's orientation (Fig. 1). The diffraction signal recorded in one frame is therefore highly incomplete [Fig. 2(a)]. Because crystals have a finite size and some extent of lattice disorder, and the spectral distribution of the X-FEL beam is limited, the recorded intensities are in general, smaller—by factors that are orientation dependent—than the corresponding diffraction intensities from an infinite and perfectly ordered crystal [Fig. 2(b)]. This effect is commonly referred to as partiality.^5^ Variations in crystal size and beam fluence distribution can be accounted for by estimating and applying frame-related scale factors. Such estimates can be computed using standard SFX software.^6^ The uncertainty in timing between pump and probe pulses (timing jitter)^7,8^ and photon counting errors^9^ are other factors that affect time-resolved serial crystallography data.

To retrieve a complete set of reciprocal-space intensities and mitigate the effects of partiality, a binning-and-merging procedure is routinely adopted (see, for example, Refs. 12–18). This approach involves dividing the pump–probe delay window into time bins, and merging the measurements by averaging the equivalent reflections within each bin. The number of frames required in the time-binning approach is commonly of the order of the tens of thousands for each time point, often leading to broad bins and a consequent deterioration of the timing information.

Here, we analyze alternative strategies to the binning-and-merging approach, with the purpose of improving the quality of the reconstructed dynamics and limiting information losses. We specifically tackle the issues of data incompleteness and partiality. To demonstrate our findings while isolating these effects, we employ a synthetic dataset. We present a new method, which we call low-pass spectral analysis (LPSA), to extract accurate dynamics from extremely incomplete and partial data. We compare the performance of LPSA, in terms of reconstruction quality and computational effort, to that of time binning as well as a range of other dynamics-retrieval techniques.

## II. OUTLINE OF THE PROBLEM

Consider m reciprocal lattice points in the resolution range of interest. Let $x_{i}\geq 0$ be the diffraction intensity related to the lattice point i. The m-tuple $x={\left(x_{1},\ldots ,x_{m}\right)}^{T}$ for a given set of non-negative numbers $x_{1},\ldots ,x_{m}$ may be viewed as a possible diffraction pattern. If the physical system giving rise to the diffraction pattern undergoes dynamical evolution, then, in the absence of stochasticity, the dynamics are governed by a differential equation with respect to time. This implies that the associated diffraction pattern as a function of time x(t) must be at least singly differentiable with respect to t. Hence, in the immediate vicinity of any time point $t_{0}$, we can approximate x(t) as follows:

$$x(t)∼x(t_{0})+\dot{x}(t_{0})(t-t_{0}).$$ (1)

This means that locally, the x(t) points lie on a straight line. Local linearity renders x(t) a one-dimensional manifold.^19^

For a given orthonormal basis of ${\mathbb{R}}^{m}$, $\left\{u_{1},\ldots ,u_{m}\right\}$, the system's trajectory in reciprocal space can be expanded as follows:

$$x(t)=\sum_{j=1}^{m}\alpha_{j}(t)u_{j},$$ (2)

with the expansion coefficients,

$$\alpha_{j}(t)=u_{j}^{T}x(t).$$ (3)

The basis vectors associated with nonnegligible expansion coefficients span the linear subspace of ${\mathbb{R}}^{m}$ explored by the trajectory x(t). As a consequence of experimental reality, the one-dimensional manifold that underlies the system's dynamical evolution may be completely unrecognizable. In practice, the stochastic effects of incompleteness and partiality alter the trajectory of the system in data space and artificially increase the apparent dimension of the subspace explored by the dynamics.

The task at hand is then twofold. First, we have to retrieve the one-dimensional manifold described by the dynamical system, that is, mitigate the stochastic effects introduced by data incompleteness and partiality. Second, we need to identify the linear space of minimal dimension in which the recovered manifold can be embedded. There are various strategies to accomplish the first of our tasks, which will be detailed in Secs. III and V. Subsequent singular value decomposition (SVD)^20,21^ allows to identify the linear subspace explored by the system.

Since the recovered dynamics are expected to describe a locally linear manifold, to help the analysis of our results, we introduce a measure of the deviation from local linearity. We denote with $\left\{x_{j}\right\}$ the sequence of time-ordered data vectors related to the time points $\left\{t_{j}\right\}$, with $x_{j}=x(t_{j})$. From any pair of temporally neighboring points, $x_{j-1}$ and $x_{j}$, we can construct a local linear approximation to x(t), which we call $x^{\left(j\right)}(t)$,

$$x^{\left(j\right)}(t)=x_{j-1}+\frac{t-t_{j-1}}{t_{j}-t_{j-1}}(x_{j}-x_{j-1}).$$ (4)

Local linearity implies that the two immediate temporal neighbors of $x_{j-1}$ and $x_{j}$, i.e., $x_{j-2}$ and $x_{j+1}$, lie close to the points $x^{\left(j\right)}(t_{j-2})$ and $x^{\left(j\right)}(t_{j+1})$, respectively. We, therefore, define

$$L^{\left(j\right)}=\frac{1}{2}\left[\left|x_{j-2}-x^{\left(j\right)}(t_{j-2})\right|+\left|x_{j+1}-x^{\left(j\right)}(t_{j+1})\right|\right].$$ (5)

The average over all $L^{\left(j\right)}$ represents our measure of deviation from local linearity L.

## III. LOW-PASS SPECTRAL ANALYSIS

We map the incomplete data to a sparse-matrix representation, that is, we set any unmeasured component of x(t) equal to zero.^22^ With this choice, the fundamental issue that we need to address and mitigate is the stochastic weighting (by factors comprised between zero and one) of the underlying intensities, introduced by sparsity and partiality. To alleviate the effects of randomness, we project the data onto the subspace spanned by a set of trigonometric functions. The frequencies of these functions are defined by integer multiples of the first harmonic corresponding to one period of oscillation in the time range covered by the (time-lagged embedded) data points and up to a certain cutoff. This procedure effectively removes undesired high-frequency features and allows to recover the system's underlying trajectory in data space. Time-lagged embedding of the data^23–25^ can be performed prior to subspace projection, to improve the stability of the reconstructed signal, but at an increased computational cost (Secs. IV and VI). Subsequent modal decomposition allows to represent the dynamical evolution of the system in the subspace of minimal dimension. We present the details of the method hereafter.

### A. Time-lagged embedding

For S sampled time points, the columns of $x\in {\mathbb{R}}^{m\times S}$ give a discretized representation of the trajectory of interest x(t). Hence, for a given time point $t_{j}$, $x_{j}=x(t_{j})$, where $x_{j}$ is the jth column of x. With the values of missing entries set equal to zero, x is typically highly sparse. The time-lagged embedding procedure, with concatenation parameter q, consists in the delayed-coordinate mapping defined by

$$x(t_{j})\;\mapsto \;X(t_{j})=\left(\begin{array}{c} x_{j}\\ x_{j-1}\\ \vdots \\ x_{j-q+1} \end{array}\right).$$ (6)

### B. Low-pass filtering

With the purpose of denoising the data by removal of the high-frequency components, and given the (time-lagged embedded) data $X\in {\mathbb{R}}^{mq\times s}$, with s=S−q+1, we define a time-domain projector $\Phi \Phi^{T}\in {\mathbb{R}}^{s\times s}$, with $\Phi \in {\mathbb{R}}^{s\times k}$. We consider the fundamental oscillation period T=2π/ω corresponding to the time window spanned by the ensemble of the time-lagged embedded points. We define $\Psi =(\psi_{1},\ldots ,\psi_{k})$, where $\psi_{j}$ is the jth column of Ψ. The matrix entries are obtained by sampling a series of trigonometric functions at discrete time points as follows:

$$\psi_{i,2j}=\text{cos}\;(j\omega t_{i}),$$ (7)

$$\psi_{i,2j+1}=\text{sin}\;(j\omega t_{i}),$$ (8)

for i=1,…,s; $j=1,\ldots ,j_{\text{max}}$ and $\psi_{1}$ is a constant vector. The columns of $\Phi ,\;(\phi_{1},\ldots ,\phi_{k})$, are obtained by orthonormalization of the vectors $\left(\psi_{1},\ldots ,\psi_{k}\right)$ to fulfill the condition,

$$\Phi^{T}\Phi =I_{k\times k},$$ (9)

with $k=2j_{\text{max}}+1$. Only if k equals s,

$$\Phi \Phi^{T}=I_{s\times s},$$ (10)

holds. Typical choices of k satisfy k≪s to (i) remove the undesired high-frequency features and (ii) make the subsequent calculations more affordable. The low-pass filtered data $X\Phi \Phi^{T}$ retain frequency components up to the cutoff $j_{\text{max}}\omega$.

### C. Modal decomposition

The linear subspace of ${\mathbb{R}}^{mq}$ explored by the system's dynamics can be identified by modal decomposition of the linear mapping $A\in {\mathbb{R}}^{mq\times k}$ given by the subspace projection,

A=XΦ. (11)

The SVD of A is

$$A=U\Sigma V^{T},$$ (12)

with $U\in {\mathbb{R}}^{mq\times r},\;\Sigma \in {\mathbb{R}}^{r\times r},\;V\in {\mathbb{R}}^{k\times r}$, and r≤k is the rank of A. Using $U=(u_{1},\ldots ,u_{r}),\;\Sigma_{ij}=\sigma_{i}\delta_{ij}$, and $V=(v_{1},\ldots ,v_{r})$, with $u_{i}$ and $v_{i}$ columns of U and V, respectively, the SVD gives the modal decomposition,

$$A=\sum_{i=1}^{r}\sigma_{i}u_{i}v_{i}^{T}.$$ (13)

### D. Reconstruction in time-lagged embedding space

The reconstructed time-lagged embedded data points are

$$\tilde{X}=A\Phi^{T}∼\sum_{i=1}^{\tilde{r}}\sigma_{i}u_{i}{\left(\Phi v_{i}\right)}^{T},$$ (14)

where only $\tilde{r}\leq r$ dominant modes are retained.

### E. Signal retrieval in data space

The reconstructed time-lagged embedded points $\tilde{X}(t_{i})$ have the structure described in Eq. (6). Hence, the data point $\tilde{x}(t)$ can be retrieved by averaging the q reconstructed copies extracted from time-lagged embedded vectors in the range from $\tilde{X}(t_{i})$ to $\tilde{X}(t_{i+q-1})$.

## IV. RESULTS OF LPSA

**FIG. 3.**
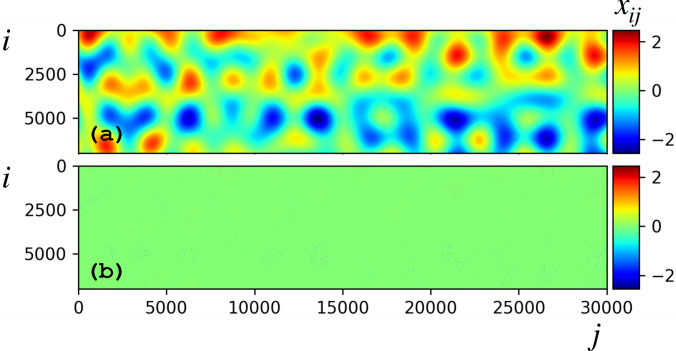
(a) Underlying dynamics x(t) [Eq. (15)], with $x_{\mathrm{ij}}$ the ith component of data vector $x_{j}=x(t_{j})$. (b) Incomplete and partial input data. Missing entries are assigned to zero values generating a sparse input data matrix.

To investigate the capabilities of LPSA and compare it to other dynamics retrieval methods, we employ the synthetic model,

$$\begin{array}{c} x(t)=(1-e^{\left(-t/t_{c}\right)})[A+B\;\text{cos}\;(3\omega t)+C\;\text{sin}\;(10\omega t)]\\ +e^{\left(-t/t_{c}\right)}[D+E\;\text{sin}\;(7\omega t)+F\;\text{sin}\;(11\omega t+\pi /10)], \end{array}$$ (15)

shown in Fig. 3(a), with $t_{c}$ corresponding to the middle of the time interval considered, A, B, C, D, E and F noncollinear vectors $\in {\mathbb{R}}^{m}$, with components,

$$\begin{array}{c} A_{i}=\text{cos}\;[0.6\chi i],\\ B_{i}=\text{sin}\;[3.0\chi i+\pi /5],\\ C_{i}=\text{sin}\;[0.8\chi i+\pi /7],\\ D_{i}=\text{cos}\;[2.1\chi i],\\ E_{i}=\text{cos}\;[1.2\chi i+\pi /10],\\ F_{i}=\text{sin}\;[1.8\chi i+\pi /11], \end{array}$$ (16)

for i=1,…,m and χ=2π/m. Fundamental molecular physics dictates that, particularly on ultrafast time scales, structural dynamics are dominated by vibrations and, occasionally, quasi-irreversible transitions between local minima of the respective potential energy surfaces. Our model [Eq. (15)] is designed to reflect both of these effects. To mimic the extent of incompleteness affecting TR-SFX datasets, we set equal to zero 98.2% of the $x_{i}(t)$ values, chosen at random (and matching the incompleteness of the dataset in Ref. 16). In addition, we multiply each data point $x_{i}(t)$ by a random number extracted from a constant distribution between zero and one [Fig. 3(b)], to model data partiality. Because the signal is generated by a linear combination of six linearly independent vectors, the dimension of the linear subspace of ${\mathbb{R}}^{m}$ explored by the underlying dynamics is six. However, the dimension of the subspace of ${\mathbb{R}}^{qm}$ explored by the time-lagged embedded data manifold is not constrained to six, which rather represents a lower limit.

We process the sparse and partial dataset by LPSA and measure the quality of the retrieved signal as a function of the number of modes $\tilde{r}$ employed in the reconstruction [Eq. (14)]. We examine the evolution of the results as we vary the two parameters involved: the concatenation parameter q and the cutoff frequency $j_{\text{max}}$. The quality of the reconstructed signal is quantified by calculating the linear correlation coefficient to the underlying dynamics from Eq. (15). Since this metric is generally unavailable, we analyze the corresponding evolution of two indicators that can be used to guide the choice of the number of modes to be employed in the reconstruction. The singular value spectrum of the matrix A shows the relative weight of the terms of the modal decomposition in Eq. (13). We expect noise terms to have a relatively low weight. In addition, we compute a measure of the deviation from local linearity of the reconstructed signal, which we call L (see Sec. II). We expect the retrieved dynamics to deviate significantly from local linearity when the number of modes employed exceeds the optimal one, and noise from the input data is reintroduced in the reconstructed signal.

**FIG. 4.**
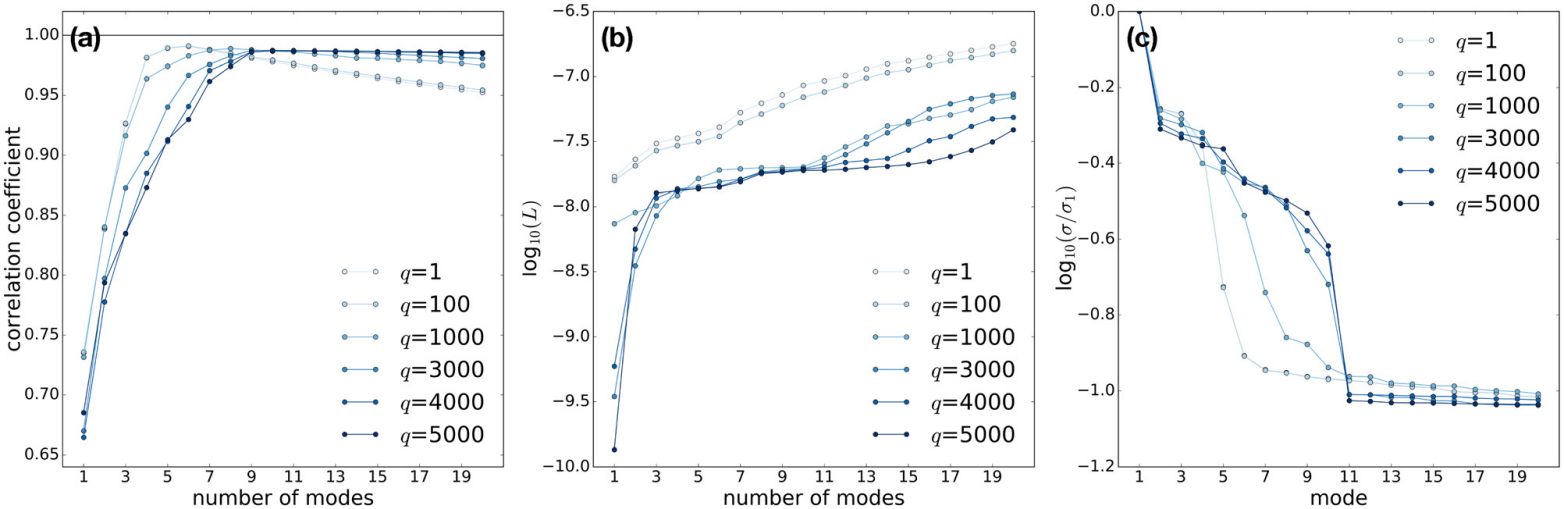
LPSA of the sparse and partial dataset shown in Fig. 3(b), for various values of q, and with $j_{\text{max}}=100.$ (a) Linear correlation coefficient between the reconstructed signal and the underlying dynamics. (b) Measure of deviation from local linearity. (c) Singular value spectrum.

Figure 4 shows the evolution of the quantities mentioned above with varying $\tilde{r}$, for various values of q and fixed $j_{\text{max}}=100$. We observe that the best linear correlation of the reconstructed signal to the ground truth is obtained with six modes and q = 1, matching our expectation that the dimension of the subspace explored by the data manifold is six [Fig. 4(a)]. We also notice that for q = 1 the correlation coefficient does not converge to its maximal value with increasing number of modes, but rather deteriorates progressively when $\tilde{r}$ increases beyond six. The choice of the number of modes is then critical to the quality of the results. Such a choice can be guided by identifying the end of the plateau section in $L(\tilde{r})$ [Fig. 4(b)]. This shows that considerable noise is added to the reconstructed signal as $\tilde{r}$ is increased beyond six modes. A concomitant sharp decline in the singular value spectrum is observed [Fig. 4(c)]. With large values of the concatenation parameter, q≫1, a robust convergence of the correlation coefficient with increasing $\tilde{r}$ is observed, at the cost of higher computational effort. The minimal number of modes required to obtain the maximal correlation is ten, in accordance with our expectation that in time-lagged embedding space, the dimension of the explored subspace can be larger than six.

**FIG. 5.**
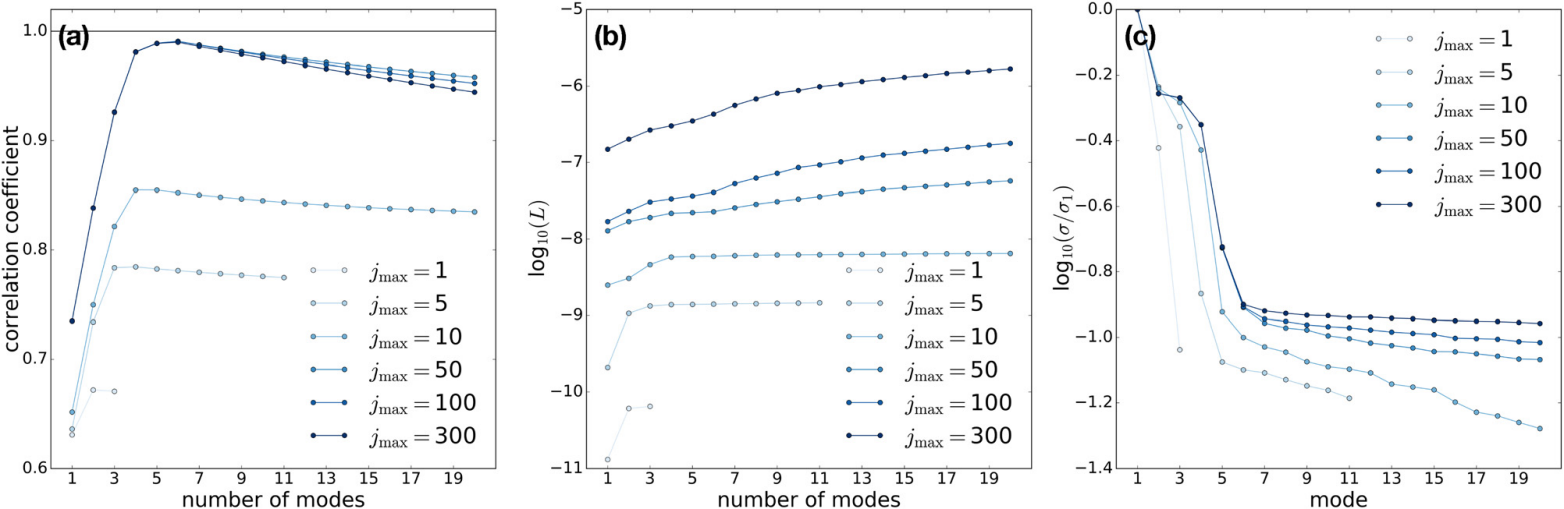
LPSA of the sparse and partial dataset shown in Fig. 3(b), for various values of $j_{\text{max}}$ and with q = 1. (a) Linear correlation coefficient between the reconstructed signal and the underlying dynamics. (b) Measure of deviation from local linearity. (c) Singular value spectrum.

**FIG. 6.**
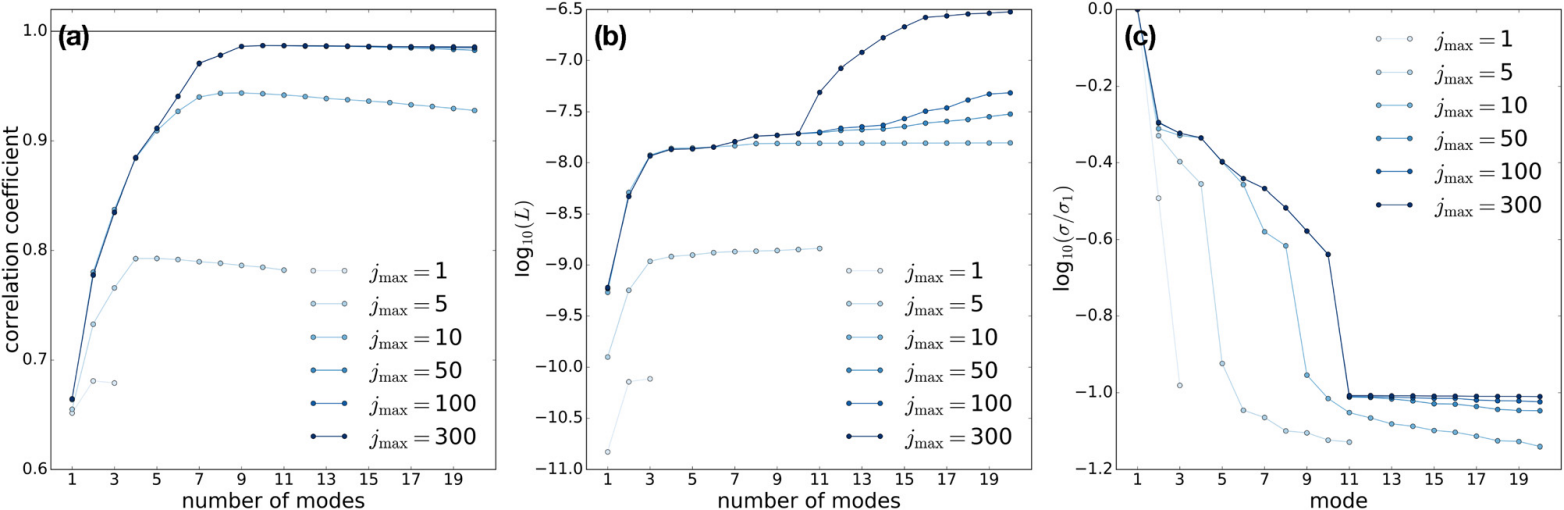
LPSA of the sparse and partial dataset shown in Fig. 3(b), for various values of $j_{\text{max}}$ and with q = 4000. (a) Linear correlation coefficient between the reconstructed signal and the underlying dynamics. (b) Measure of deviation from local linearity. (c) Singular value spectrum.

We now analyze the evolution of the results with varying $j_{\text{max}}$, for q = 1 (Fig. 5) and q = 4000 (Fig. 6). The results converge toward the optimal reconstruction with increasing $j_{\text{max}}$, with q = 1 and $\tilde{r}=6$; and with q = 4000 and $\tilde{r}\geq 10$. We observe a degradation of the reconstruction quality as the number of modes exceeds the optimal one for q = 1, but a robust convergence with increasing number of modes for q = 4000. The measure L of deviation from local linearity [Figs. 5(b) and 6(b)] and the singular value spectrum [Figs. 5(c) and 6(c)] can guide the choice of $\tilde{r}$. Relatively high noise levels in the reconstructed signal, and relatively low singular values are observed when the optimal number of modes is exceeded. In particular, with q = 4000 and sufficiently high $j_{\text{max}}$, the local linearity measure allows to detect a sharp increase in noise reconstruction beyond 10 modes, in agreement with the abrupt decline of the singular value.

## V. COMPARISON WITH OTHER DYNAMICS RETRIEVAL METHODS

### A. Singular spectrum analysis

Time-lagged embedding followed by modal decomposition by SVD is known as singular spectrum analysis (SSA).^26^ With concatenation parameter q = 1, SSA is equivalent to SVD.

### B. Nonlinear Laplacian spectral analysis

Nonlinear Laplacian spectral analysis (NLSA) was introduced in Ref. 27 and used in the context of time resolved experiments in Refs. 22 and 28. Similar to LPSA, the overarching framework is that of a subspace projection preceded by time-lagged embedding and followed by modal decomposition. The difference between the two methods resides in the choice of the subspace basis set. In NLSA, a data-driven basis set is employed, specifically a set of functions derived from the diffusion map algorithm.^29^ In this work, we propose a modified version of the diffusion map, which allows to obtain a set of orthonormal vectors to use directly as a subspace basis set. We also consider two different formulations of NLSA. In the standard version (E-NLSA), each data point's Euclidean nearest neighbors are considered. With the purpose of using all available information, we propose a procedure whereby time nearest neighbors are retained instead (T-NLSA). Here, timing information is used to guide the nearest-neighbor selection. As described in Sec. III, missing observations are set equal to zero.

#### 1. Distance calculation

Euclidean distances between highly sparse time-lagged embedded vectors are calculated by retaining only common terms (i.e., the set of reflections that are present in both time-lagged embedded vectors), and are normalized by the number of retained components. This approach appears to better represent underlying distances (those pertaining to the underlying dynamics), compared to distances calculated by including all components.

#### 2. Diffusion map

We use the diffusion map kernel,

$$K_{ij}=\text{exp}\;[-D_{ij}^{2}/(2\epsilon )],$$ (17)

as a measure of similarity between time-lagged embedded points. In this expression, $D_{ij}$ are Euclidean distances in ${\mathbb{R}}^{mq}$ and ϵ refers to the extent of the local neighborhood. An estimate of this parameter is obtained as described in Ref. 30. For each time-lagged embedded point, b≪s nearest neighbors are considered. In standard NLSA, b Euclidean nearest neighbors are retained (E-NLSA). Alternatively, we consider b nearest neighbors in time (T-NLSA). After symmetrization, the diffusion kernel is normalized to consider local densities:^31^

$${\tilde{K}}_{ij}=\frac{K_{ij}}{\sum_{i}K_{ij}\sum_{j}K_{ij}}.$$ (18)

With $Q_{i}=\sum_{j}{\tilde{K}}_{ij}$ and $Q_{ij}=Q_{i}\delta_{ij}$, we define the diagonal and positive-definite matrix Q. We solve the eigenvalue problem,

WΦ=ΦΛ, (19)

for the real and symmetric matrix,

$$W=Q^{-1/2}\tilde{K}Q^{-1/2}.$$ (20)

The orthonormal eigenvectors Φ are closely related to the (in general, nonorthogonal) eigenvectors of the probability matrix,

$$P=Q^{-1}\tilde{K},$$ (21)

obtained by row-normalization of $\tilde{K}$.

#### 3. Modal decomposition and reconstruction

We use a subset of the orthonormal eigenvectors Φ to construct a data-driven projector to a subspace of ${\mathbb{R}}^{s}$. A number l of eigenvectors, related to the eigenvalues $\Lambda_{ii}$ with largest absolute value, are retained and used as a basis set for the subspace projection, analogous to Eq. (11). Modal decomposition and signal reconstruction are carried out as described in Sec. III.

### C. Time binning

To compare the dynamics-retrieval results to those from time binning and merging, we compute the running average of x(t) for various values of the time bin size.

## VI. DISCUSSION

**FIG. 7.**
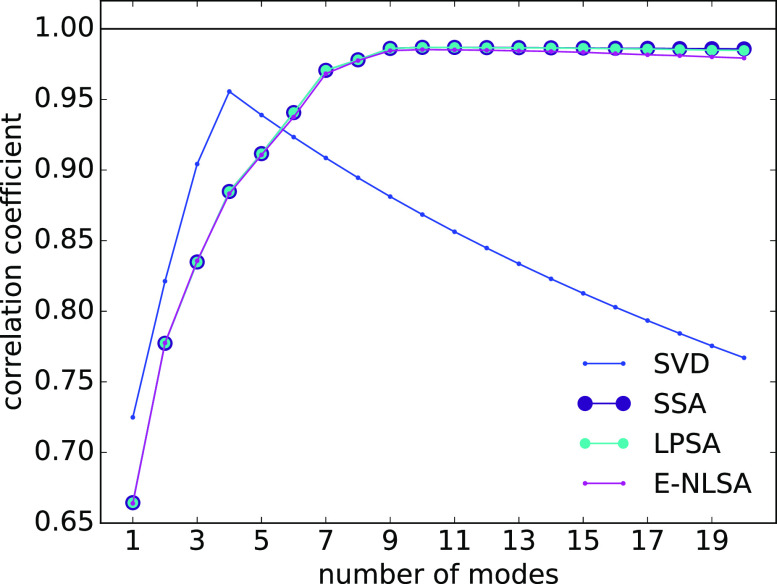
Comparison between SVD, SSA ( q=4000), LPSA ( $q=4000,j_{\text{max}}=100$), E-NLSA ( $q=4000,b=3000,\;{\text{log}}_{10}\epsilon =1.0,l=50$). Linear correlation between the reconstructed dynamics from sparse and partial input data and the underlying dynamics.

We compare the performance of LPSA to that of the methods presented in Sec. V on the task of reconstructing the synthetic signal presented in Sec. IV [Eq. (15)], from input data with extreme incompleteness and partiality. Figure 7 shows the evolution of the linear correlation between the recovered signal and the underlying dynamics, as a function of the number of modes employed, for the various techniques considered.

With an increasing number of modes, the reconstructed signal from pure SVD reproduces more and more closely the sparse and partial input data. A maximum in the correlation between the reconstructed signal and the underlying dynamics is observed with four modes. As the number of modes exceeds four, the correlation deteriorates. The maximal correlation achievable by SVD lies well below that from concatenation-based or projection-based methods. By including a time-lagged embedding step (SSA), the reconstruction achieves optimal quality with ten modes, and shows a robust convergence with increasing number of modes. The drawback resides in the large size of the matrix $X\in {\mathbb{R}}^{mq\times s}$ to be singular-value decomposed.

NLSA produces excellent reconstruction results, similar to SSA. The subspace projection allows to reduce the size of the matrix to be singular-value decomposed ( $A\in {\mathbb{R}}^{mq\times l}$, with l≪s). However, the computation of data-driven subspace basis vectors is expensive, as it involves the calculation of $s^{2}$ (in E-NLSA) Euclidean distances between tuples in ${\mathbb{R}}^{mq}$, and the eigendecomposition of the large (but sparse) matrix $W\in {\mathbb{R}}^{s\times s}$. A specific drawback of NLSA is that the parameter space to be considered is four dimensional (q,b,ϵ,l).

**FIG. 8.**
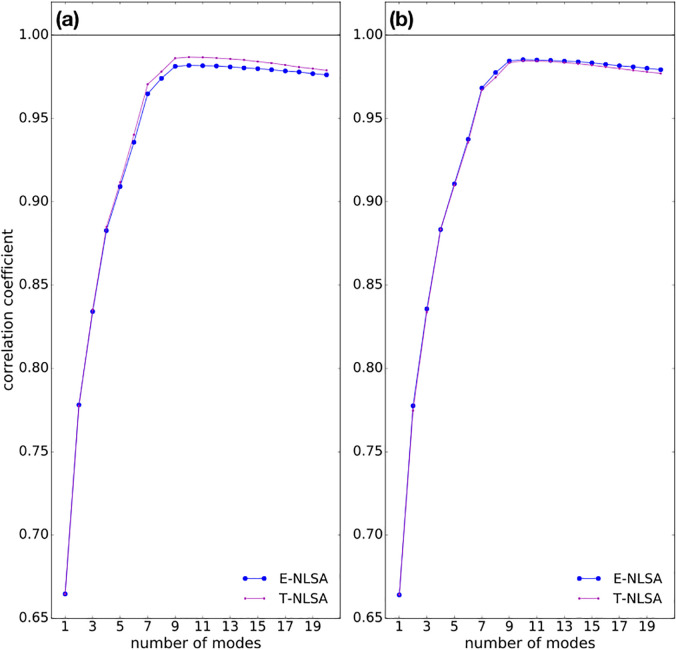
Comparison between E-NLSA and T-NLSA. Linear correlation between the reconstructed signal and the underlying dynamics as a function of the number of modes employed. The NLSA was computed with concatenation parameter q=4000, subspace dimension l=50, neighborhood size $\;{\text{log}}_{10}\epsilon =1.0$, nearest-neighbor number (a) b=1500 and (b) b=3000.

We compare the results from E-NLSA and T-NLSA, for b = 1500 and b = 3000. Figure 8 shows that the best reconstruction is obtained with T-NLSA and b = 1500. This is due to the fact that T-NLSA effectively uses the time order of the input data as prior knowledge to guide the choice of the nearest neighbors. In addition, compared to E-NLSA, T-NLSA presents the advantage that only bs, rather than $s^{2}$, Euclidean distances must be computed. The use of a data-driven subspace basis may be important when dealing with chaotic dynamical systems, whereby the underlying dynamics explores a truly high-dimensional subspace of ${\mathbb{R}}^{mq}$. NLSA appears in this case to effectively provide a low-rank representation of the dynamics, where SSA fails to do so.^27^ Data-driven basis functions were found to approximate a set of periodic functions at large values of the concatenation parameter.^32^

The dynamics retrieval from LPSA is excellent, as shown in Fig. 7. Similar to NLSA, the subspace projection allows to reduce the size of the matrix to be singular-value decomposed ( $A\in {\mathbb{R}}^{mq\times k}$, with $k=2j_{\text{max}}+1\ll s$). In contrast to NLSA, the computation of the subspace basis set is inexpensive in LPSA. In addition, LPSA only involves two parameters $\left(q,j_{\text{max}}\right)$, which represents a major practical advantage compared to NLSA. LPSA involves particularly cheap computations when no time-lagged embedding is performed ( q=1). While excellent results can be achieved in this case, it is important to consider that the number of employed modes plays a major role in determining the quality of the reconstruction, as convergence with respect to the number of modes cannot be assured. In this case, the singular value spectrum, and the deviation from local linearity measured by the indicator L, can be used to guide the choice of the number of modes.

Typical values in TR-SFX applications are $S∼10^{5},\;m∼10^{4},q∼10^{4}$, and $s∼10^{5}$. In this context, SSA mandates the SVD of a 10^13^-element matrix. By contrast, projection-based methods involve the SVD of a much smaller matrix. With the number of basis vectors typically ranging between 10^1^ and 10^2^, A is substantially smaller than X in both NLSA and LPSA TR-SFX applications. However, the calculation of data-driven basis vectors for the NLSA subspace projection is computationally expensive. To this end, the calculation of distances between time-lagged embedded vectors is particularly challenging. To give an example, the analysis of a 10^5^-frame dataset, with $m∼10^{4}$ and $q∼10^{4}$, involves the computation of $∼10^{10}$ Euclidean distances between 10^8^-element tuples. In addition, to obtain data-driven subspace basis vectors, a (sparse) 10^10^-element matrix must be eigendecomposed. In this respect, LPSA presents the practical advantage that the subspace basis vectors can be readily computed as a set of orthonormalized trigonometric functions.

**FIG. 9.**
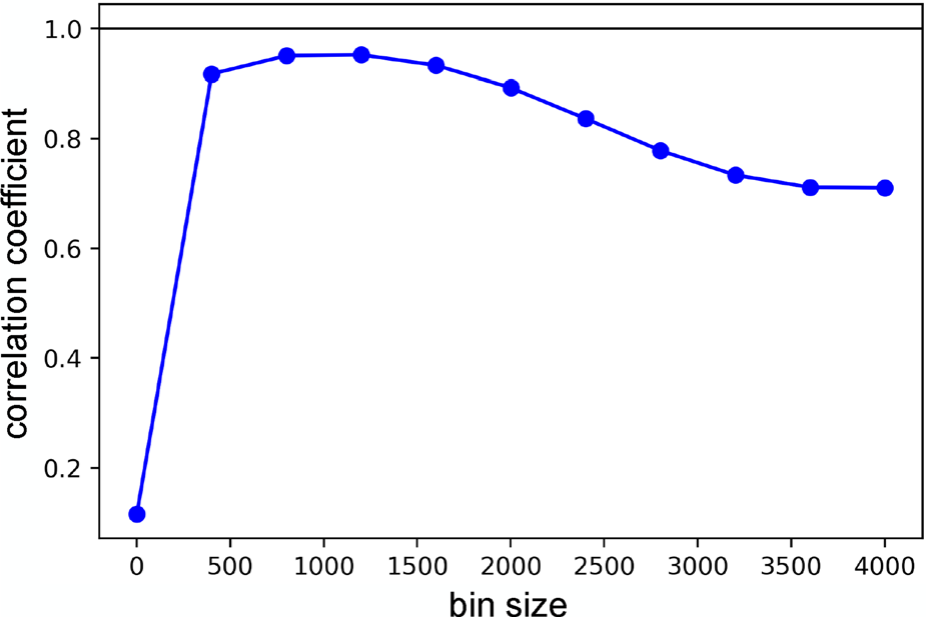
Linear correlation between the binned and merged signal obtained by computing the running average of x(t), and the underlying dynamics, as a function of the bin size employed.

**FIG. 10.**
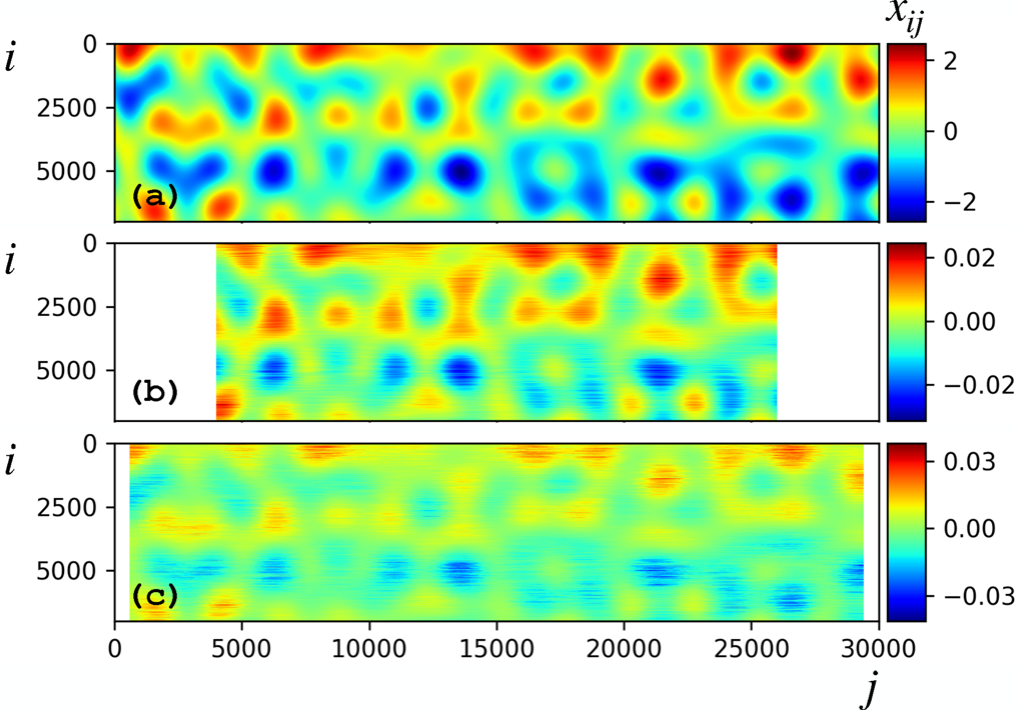
(a) Underlying dynamics x(t) [Eq. (15)]. (b) Reconstruction by LPSA with q=4000, $j_{\text{max}}=100$ and 10 modes. The correlation coefficient to the underlying signal is 0.9869. (c) Reconstruction by time-binning and merging with bins comprising 1201 frames. The correlation coefficient to the underlying signal is 0.9519.

Finally, we compare our results to those from time binning and merging, which is to date the most commonly used technique to analyze TR-SFX data. Figure 9 shows the linear correlation between the binned and merged signal and the underlying dynamics, as a function of the size of the time bins. The maximal correlation achieved is 0.952, below the value of 0.987 obtained by LPSA. The difference in the quality of the reconstruction can be appreciated by comparing the binned and merged signal in Fig. 10(c) to the LPSA results in Fig. 10(b). It should be emphasized that, in the standard binning-and-merging approach, there is no intrinsic way to ensure whether the width of each bin has been selected optimally. Here, we present the best-case scenario, in which we choose the optimal bin size by maximizing the correlation with the benchmark, which is generally not available. By contrast, LPSA parameters can be optimized based on the deviation from local linearity and the singular value spectrum, i.e., indicators that do not depend on *a priori* knowledge of the ground truth.

## VII. CONCLUSIONS

We have presented a new approach to retrieving dynamical information from highly incomplete and partial data, of the type obtained by TR-SFX experiments. This approach, which we call LPSA, allows an improved signal reconstruction, compared to time binning and merging, which is to date the most common procedure to gain dynamical insight from TR-SFX data. We have also shown that, while achieving the same reconstruction quality as other sophisticated dynamics retrieval techniques (SSA, NLSA), LPSA presents practical advantages, in particular concerning the computational cost of the algorithm, and the number of parameters to be optimized. While being developed in the context of TR-SFX data analysis, LPSA is a general tool for the analysis of time series affected by stochastic weighting and incompleteness, which could be employed in a diverse range of applications in science and engineering.

## Data Availability

The data that support the findings of this study are available within the article.
